# Molecular phylogenetic analyses based on the complete plastid genomes and nuclear sequences reveal *Daphne* (Thymelaeaceae) to be non-monophyletic as current circumscription

**DOI:** 10.1016/j.pld.2021.11.001

**Published:** 2021-11-11

**Authors:** Shiou Yih Lee, Ke-Wang Xu, Cui-Ying Huang, Jung-Hyun Lee, Wen-Bo Liao, Yong-Hong Zhang, Qiang Fan

**Affiliations:** a State Key Laboratory of Biocontrol and Guangdong Provincial Key Laboratory of Plant Resources, School of Life Sciences, Sun Yat-sen University, 510275, Guangzhou, China; b Co-Innovation Center for Sustainable Forestry in Southern China, College of Biology and the Environment, Key Laboratory of State Forestry and Grassland Administration on Subtropical Forest Biodiversity Conservation, Nanjing Forestry University, 219937, Nanjing, China; c Department of Biology Education, Chonnam National University, 61186, Gwangju, Republic of Korea; d School of Life Sciences, Yunnan Normal University, 650001, Kunming, China; e Faculty of Health and Life Sciences, INTI International University, 71800, Nilai, Malaysia

**Keywords:** Daphneae, Comparative plastome analysis, Internal transcribed spacer region, Polyphyletic relationship, *Wikstroemia*

## Abstract

The diverse members of the genus *Daphne* are prized for their fragrant flowers. Despite being promising ornamental plants in many countries, genetic information of *Daphne* is scarce. In this study, the plastomes of four species and one variety of *Daphne* were sequenced and analyzed. The plastomes were typical and contained a pair of inverted repeat (IR) regions that separated the large single-copy (LSC) region from the small single-copy (SSC) region. With a length ranging from 132,869 bp (*D. genkwa*) to 174,773 bp (*D. championii*), 106 to 141 genes were predicted. Comparative plastome analysis of the newly sequenced plastomes with four publicly available *Daphne* plastomes identified an expansion of the IRs, sequence variations, and mutational hotspots. Phylogenetic analyses indicated that the genus *Daphne* in its current circumscription is polyphyletic. *Daphne genkwa* was nested within the genus *Wikstroemia*, while *D. championii* was well resolved as sister to *Edgeworthia*. These findings concurred with results from our study that used nuclear ribosomal internal transcribed spacer sequence data. The conflicts on the molecular placement of *D. championii* and *D. genkwa* and the present taxonomic classification in *Daphne* suggest that a new intergeneric classification system of Daphneae warrants consideration.

## Highlights

• Novel plastome sequences of four species and one variety of *Daphne* were reported.
• Variations in gene content and arrangement in the plastomes of the sample species of *Daphne* were identified.
• Plastome and ITS sequences indicate that *Daphne* is not monophyletic.

## 1 Introduction

The family Thymelaeaceae comprises 45 genera and about 800 species widely distributed in both temperate and tropical regions (Herber, 2003). Recent taxonomic work on Thymelaeaceae has divided the family into two subfamilies: Octolepidoideae and Thymelaeoideae (Herber, 2003). The latter includes the tribes Aquilarieae, Daphneae, and Synandrodaphneae. The tribe Daphneae, synonym Thymelaeoideae *sensu* Domke (1934), is further subdivided into four groups: the Daphne, Gnidia, Linostoma, and Phaleria groups. Members of the Daphne group can be easily distinguished by their lack of interxylary phloem; also, no genera except *Diarthron* Turcz. have unarticulated floral tubes. Fourteen genera have been recorded in the Daphne group: *Daphne* L., *Daphnopsis* Mart. & Zucc., *Diarthron*, *Dirca* L., *Edgeworthia* Meisn., *Funifera* Leandro ex C.A. Mey., *Goodallia* Benth., *Lagetta* Juss., *Ovidia* Meisn., *Rhamnoneuron* Gilg., *Schoenobiblus* Mart. & Zucc., *Stellera* L., *Thymelaea* Mill., and *Wikstroemia* Endl. (Herber, 2003).

The genus *Daphne* is one of the largest genera in the Daphne group, comprising ca. 95 species of well-known ornamental plants distributed in Eurasian and North African (Herber, 2003; Wang et al., 2007a). Thus far, the International Union for Conservation of Nature (IUCN) has only addressed the conservation status of six *Daphne* species, classifying *Daphne rodriguezii* Texidor and *D. sophia* Kolenicz. as “Endangered”, *D. ludlowii* D.G. Long & Rae and *D. petraea* Leyb. as “Least Concern”, and *D. altaica* Pall. and *D. arbuscular* Celak. as “Data Deficient” in the IUCN Red List (IUCN, 2020). Because *Daphne* spp. are commonly cultivated in parks and gardens (Brickell and Mathew, 1998) and are known to have medicinal properties (Sovrlić and Manojlović, 2017), most studies of this genus have focused on either horticultural or medicinal properties. Although genetic studies have been conducted on *Daphne jezoensis* Maxim., *D. laureola* L., and *D. rodriguezii* (Alonso and Herrera, 2011; Castilla et al., 2012; Kameyama and Hirao, 2014; García-Verdugo et al., 2019), species demarcation in *Daphne* is based solely on floral characteristics (Wang et al., 2007a). However, *Daphne* species are morphologically similar, suggesting that molecular approaches are required to elucidate their taxonomy.

The circumscription of *Daphne*, as well as its phylogenetic relationship with allied genera, has long been controversial. For instance, the boundary between *Daphne* and *Wikstroemia* remains in dispute (Hamaya, 1963; Wang et al., 2007a), prompting some to propose transferring *Wikstroemia* into *Daphne* and further treating it as a subgenus (Halda, 1999, Halda, 2001; Wang et al., 2007a; Zhang et al., 2007). The ambiguity in phylogenetic relationships is largely because the features traditionally used to distinguish species (i.e., the shape of hypogynous disc, the type of fruit, and the leaf arrangement) are exhibited across the genus (Domke, 1934; Hamaya, 1963; Halda, 1999; Wang et al., 2007b), and because of the lack of molecular resources to test phylogenetic inferences. Although molecular studies have been conducted on Thymelaeaceae at the genus-level, these studies have mostly focused on the subfamily Thymelaeoideae, with particular emphasis on a few genera (*Gnidia* L., *Passerina* L. mainly from South Africa, *Thymelaea* from circum-Mediterranean area, and *Pimelea* Banks ex Sol. from the Asia–Pacific region) (Van der Bank et al., 2002; Galicia-Herbada, 2006; Beaumont et al., 2009; APG IV, 2016; Foster et al., 2018). Such inadequate taxonomic sampling has failed to yield insights into phylogenetic relationships within *Daphne* or between *Daphne* and other related genera (Galicia-Herbada, 2006; Wang et al., 2007b; Beaumont et al., 2009; Zhang et al., 2010).

The plastid genome, or plastome, is an ideal tool for molecular taxonomic studies. Plastomes are small, haploid, inherited uniparentally, possess low nucleotide substitution rates, and have highly conserved sequences. Nuclear ribosomal internal transcribed spacer (nrITS) regions, which unlike chloroplast DNA are inherited biparentally, are also known to be useful in assessing genetic variation and reconstructing phylogenetic relationships between closely related species (Neves and Forrest, 2011). For members of Thymelaeaceae, publicly available nrITS sequences far outnumber plastome sequences, likely because nrITS regions are easier to amplify (Alvarez and Wendel, 2003). Used in conjunction, plastid genomes and nrITS sequences promise to advance our understanding of the phylogenetic relationships of *Daphne* in Thymelaeaceae.

To test the monophyly of *Daphne*, we first assembled complete plastomes of five *Daphne* taxa, namely *D. championii*, *D. genkwa* Siebold & Zucc., *D. kiusiana* var. *atrocaulis* (Rehder) F. Maek., *D. odora* Thunb., and *D. papyracea* Wall. ex G. Don. Although these five *Daphne* species grow in the wild and are domesticated for medicinal or ornamental purposes, their respective genetic identities still remain ambiguous. We analyzed and compared these *Daphne* plastomes to published plastomes to determine the molecular identities, genetic divergence, and phylogenetic relationships of these species. We also identified highly variable gene regions that may be useful DNA barcodes for *Daphne* species. The findings of this study will provide future reference for taxonomic studies of *Daphne* and help elucidate the taxonomy of Thymelaeaceae.

## 2 Material and methods

### 2.1 Plant materials and DNA extraction

Fresh leaves from five *Daphne* taxa were collected from natural populations and arboreta in China. Samples of *D. odora* were collected from the Germplasm Resource Nursery of Ornamental Plants of Guangzhou Institute of Forestry and Landscape Architecture in the Guangdong Province, while *D. kiusiana* var. *atrocaulis* and *D. papyracea* were collected from their natural habitat in Mount Luoxiao, Hunan Province and Mount Yunkai, Guangdong Province, respectively. *D. championii* was collected from Lianzhou, Guangdong Province, and *D. genkwa* was collected from Mount Tiantai, Hubei Province and Anqing, Anhui Province. Leaves were stored with silica gel in aluminum sealed ziplock bags until DNA extraction. The voucher specimens were deposited at the Herbarium of Sun Yat-sen University (SYS) and Herbarium of Yunnan Normal University (YNUB) (Table 1).

**Table 1:** General information and NCBI GenBank accession numbers of eight *Daphne* taxa used in this study.

Species (Sample number)	Collector name and voucher record	Number of individuals examined	Sampling location	GenBank accession numbers	
				Complete chloroplast genome	nrITS
*Daphne championii* (1–4)	Shiou Yih Lee & Xinjian Zhang, LSY-THY-4001	4	Lianzhou, Guangdong, China	MT648376	MT623676-MT623679
*Daphne genkwa*	Yonghong Zhang, SBK12.	1	Mount Tiantai, Hubei, China	MN563133	–
*Daphne genkwa* (1–4)	Shiou Yih Lee, LSY-THY-4013	4	Anqing, Anhui, China	–	MT623680-MT623683
*Daphne kiusiana* var. *kiusiana* (1–4)	Jung-Hyun Lee, LJH-GU01	4	Ryuhosan, Kumamoto, Japan	–	MT623684-MT683687
*Daphne kiusiana* var. *kiusiana* (5–8)	Jung-Hyun Lee, LJH-SP01	4	Sinpyeong goj-jawal, Jeju-do, Korea	KY991380	MT623688-MT623691
*Daphne kiusiana* var. *atrocaulis* (1–3)	Wenbo Liao, LXP6488	3	Mount Luoxiao, Hunan, China	MT627481	MT623692-MT623694
*Daphne odora* (1–4)	Shiou Yih Lee, LSY-THY-4009	4	Guangzhou Institute of Forestry and Landscape Architecture, Guangdong, China	MT627479	MT623695-MT623698
*Daphne papyracea* (1–4)	Shiou Yih Lee & Zhihui Chen, LSY-THY-4005	4	Mount Yunkai, Guangdong, China	MT627480	MT623699-MT623702

Total genomic DNA extraction was carried out using the DN15-Plant DNA Mini Kit (Aidlab, China) according to the manufacturer's protocol. The quantity and quality of the DNA extracts for next-generation and Sanger sequencing were determined using Qubit™ 4 Fluorometer (Thermo Fisher Scientific, USA) and Nanodrop™ 2000 spectrophotometer (Thermo Fisher Scientific, USA), respectively.

### 2.2 Plastid genome sequencing, assembly, and annotation

A ∼350-bp insert size genomic library was prepared using a TruSeq DNA Sample Prep Kit (Illumina, USA) and sequencing was conducted on an Illumina NovaSeq platform (Illumina, USA) to obtain 6 Gb of 150-bp pair-end reads. Adapter sequences were removed using NGSToolkit (Patel and Jain, 2012), and the raw reads were fed into the NOVOPlasty 2.7.2 pipeline (Dierckxsens et al., 2017) for *de novo* assembly. Using the *rbc*L sequences for *Daphne papyracea* (LC527404) as the seed sequence, a single contig was obtained at the end of the process for each taxon. The assembled genomes were annotated through the online GeSeq annotation tool (Tillich et al., 2017) and manually checked for errors. The GC content was analysed using MEGA 7 (Kumar et al., 2016) and the circular map of each plastome was created in OGDRAW 1.3.1 (Greiner et al., 2019). The plastome sequences were deposited in the NCBI GenBank database under the accession numbers MT648376 (*D. championii*), MN563133 (*D. genkwa*), MT627481 (*D. kiusiana* var. *atrocaulis*), MT627479 (*D. odora*), and MT627480 (*D. papyracea*).

### 2.3 Sequence repeats, codon usage, and RNA editing site prediction

Forward, reverse, and palindromic repeat sequences were identified using REPuter (Kurtz et al., 2001), with the Hamming distance set at 3 and the minimum repeat size at 30 bp. The nucleotide sequence of each protein-coding gene in the plastome was extracted for subsequent analyses using FeatureExtract 1.2L Server (Wernersson, 2005). The relative synonymous codon usage (RSCU) for each protein-coding gene was calculated using the Codon Usage Calculator function available in the Sequence Manipulation Suite (Stothard, 2000), and the potential RNA editing sites were predicted using the PREP-Cp function available in the PREP Suite (Mower, 2009) based on default settings.

### 2.4 Genetic pairwise distance, IR border analysis, and genome comparison

The plastome sequences of four *Daphne* species (*D. kiusiana* var. *kiusiana* (KY991380), *D. giraldii* (MN080709), *D. laureola* (MN201546), and *D. tangutica* (MK455900)) were downloaded from NCBI GenBank and were included in subsequent analyses. All nine plastomes were aligned using MAFFT (Katoh et al., 2019) and genetic pairwise distance was calculated based on the Kimura 2-parameter DNA substitution model, with 1000 bootstrap replicates, using MEGA 7 (Kumar et al., 2016). Gaps and missing data in the alignment were not included in the analyses (complete deletion). The borders of the four different regions in the plastomes (large single-copy (LSC), small single-copy (SSC), and inverted repeat A and B (IRA and IRB)) of the nine *Daphne* species were plotted using IRscope to analyse the exact IR border positions and identify adjacent genes (Amiryousefi et al., 2018). The nine plastomes were also aligned and visualised using mVISTA program (Frazer et al., 2004) in Shuffle-LAGAN mode, using *D. laureola* (MN201546) as the reference genome.

### 2.5 Polymerase chain reaction (PCR) and Sanger sequencing

Three to four biological replicates from each species were selected for Sanger sequencing to obtain their nrITS sequences. PCR amplification of the nrITS region was carried out using primers ITS-p5: 5′-CCT TAT CAY TTA GAG GAA GGA G-3’ (Cheng et al., 2016) and ITS-S3R: 5′-GAC CGT TCT CCA GAC TAC AAT-3’ (Chiou et al., 2007). PCR was conducted in a final reaction volume of 20 μL, containing 10 μL of 2 × Taq PCR Starmix with loading dye (Genstar Biosolutions, China), 0.4 μM of each primer, and 20 ng of genomic DNA as template. PCR amplification was conducted in a T100™ Thermal Cycler (Bio-Rad, USA), with thermal settings programmed as follows: initial denaturation at 94 °C for 5 min; 40 cycles of 94 °C for 30 s, 55 °C for 30 s, and 72 °C for 30 s; and a final extension at 72 °C for 7 min. PCR products were sent for direct sequencing for both ends on an ABI 3730 DNA Analyzer (Applied Biosystems, USA).

### 2.6 Phylogenetic inference

Prior to phylogenetic analyses, all nine *Daphne* plastome sequences and the nrITS sequences of 39 accessions representing 17 *Daphne* taxa were aligned using MAFFT (Katoh et al., 2019), separately. Based on the findings of Lee et al. (2018), *Eucalyptus grandis* (Myrtaceae; HM347959 and AF390472) and *Gossypium hirsutum* (Malvaceae; DQ345959 and KC404827) were included as outgroups. Both alignments were trimmed using trimAl v.1.2 (Capella-Gutiérrez et al., 2009) with the gappyout method to reduce the systematic errors produced due to poor alignment. Phylogenetic analyses of plastome sequences were carried out using maximum likelihood (ML) and Bayesian inference (BI). Maximum likelihood (ML) tree analysis was conducted with RAxML (Stamatakis, 2014), available in the CIPRES Science Gateway (Miller et al., 2010), and using the general time-reversible (GTR) with gamma distribution (+G) (=GTR + G) nucleotide substitution model and 1000 bootstrap replicates for each branch node. Bayesian inference (BI) tree analysis was conducted using MrBayes (Ronquist et al., 2012), available in the CIPRES Science Gateway (Miller et al., 2010), based on default parameters, with minor adjustments: a mixed substitution type (Nst) was selected for the likelihood model and 2,000,000 generations were set for the Markov chain Monte Carlo (MCMC), with data sampling collected every 100 generations. The final tree results from both analyses were visualized using FigTree (Rambaut, 2018). Phylogenetic analyses of nrITS sequences was carried out using ML and maximum parsimony (MP) methods that are embedded in MEGA 7 (Kumar et al., 2016). The optimum DNA substitution model for the ML tree was calculated using the “Find Best DNA/Protein Model (ML)” function available in MEGA 7 and ML tree was constructed using the GTR + G with invariant included (+I) (GTR + G + I) nucleotide substitution model and 1000 bootstrap replicates for each branch node. The MP tree was constructed by means of 1000 bootstrap replicates, under the subtree-pruning-regrafting search method. Gaps and missing data were included in the construction of both trees.

### 2.7 Identification of divergence hotspot and potential DNA barcoding regions

To detect the nucleotide variability (Pi) in the *Daphne* plastomes, the plastome sequences were aligned using MAFFT (Katoh et al., 2019). Analyses on singleton variable sites and sliding windows, with 500-bp step size and 1000-bp window length, were performed on DnaSP v.5.1 (Librado and Rozas, 2009).

## 3 Results

### 3.1 Plastid genome features of five *Daphne* species

**Fig. 1:**
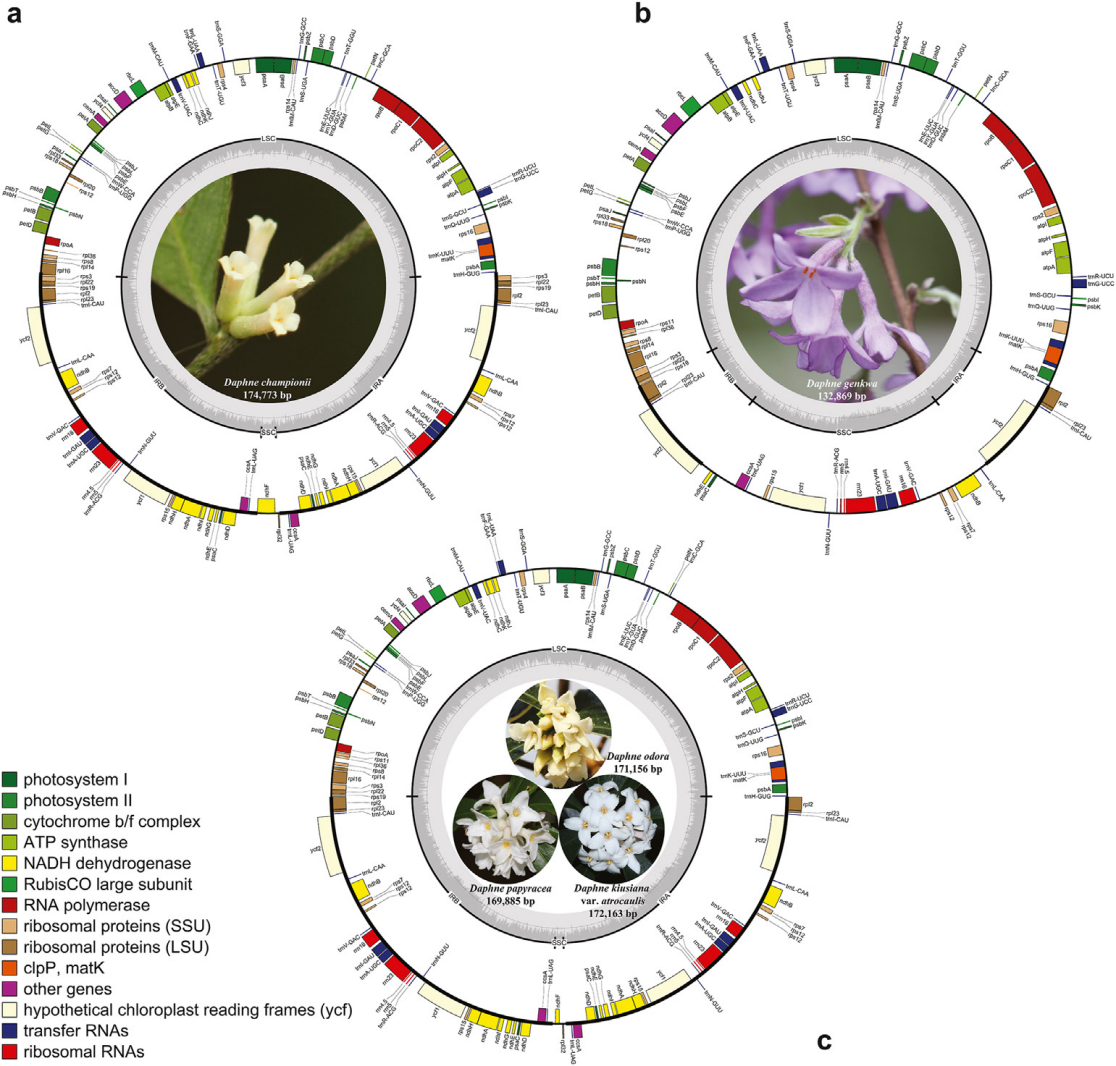
Plastid genome maps of four species and one variety in genus *Daphne*. (a) *D. championii*, (b) *D. genkwa*, (c) *D. kiusiana* var. *atrocaulis*, *D. odora*, and *D. papyracea*. Genes are color-coded according to function. Genes inside the circle are transcribed clockwise, while genes outside the circle are transcribed counterclockwise.

The total plastome sizes for the five *Daphne* taxa ranged from 132,869 (*D. genkwa*) to 174,773 bp (*D. championii*) (Fig. 1). All five plastomes exhibit a typical quadripartite structure consisting of a pair of IRs (9372–43,948 bp) that separate the LSC (84,681–85,728 bp) and SSC (1557–28,397 bp) regions. Total plastome gene number ranged from 106 (*D. genkwa)* to 141 (*D. championii)*. Plastomes included from 71 (*D. genkwa)* to 95 (*D. championii)* protein-coding genes, 31 (*D. genkwa)* to 38 (*D. championii*, *D. odora*, and *D. papyracea*) tRNA genes, and four (*D. genkwa)* or eight (*D. genkwa*, *D. kiusiana* var. *atrocaulis*, *D. odora*, and *D. papyracea*) rRNA genes. Of these genes, four are duplicated in the plastome of *D. genkwa*, while 28 are duplicated in the plastomes of *D. kiusiana* var. *atrocaulis*, *D. odora*, and *D. papyracea*, and 31 are duplicated in the plastome of *D. championii*, all in the IR regions. The GC content of the plastomes ranged from 36.3% (*D. genkwa)* to 36.8% (*D. papyracea*).

### 3.2 Large repeats analyses

**Fig. S1:**
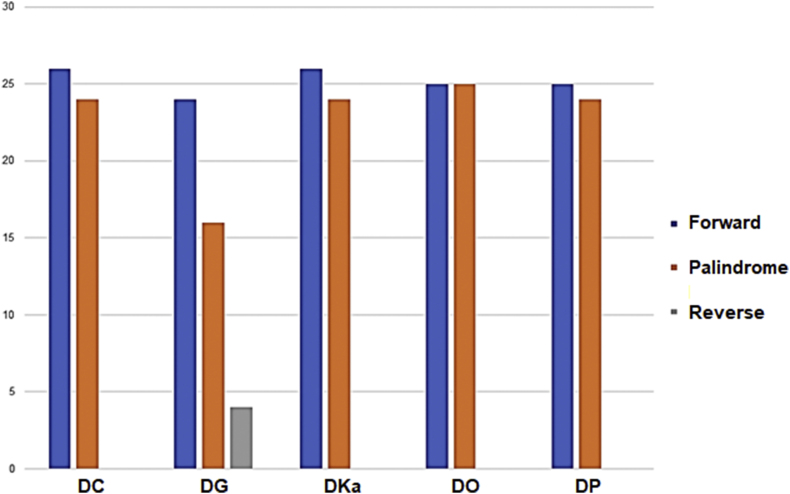
Large repeat sequence analyses of plastid genomes for five *Daphne* taxa. DC = *Daphne championii*; DG = *Daphne genkwa*; DKa = *Daphne kiusiana* var. *atrocaulis*; DO = *Daphne odora*; DP = *Daphne papyracea.*

Large repeats were detected in the plastomes of all five *Daphne* taxa, including between 24 (*D. genkwa*) and 26 (*D. championii* and *D. kiusiana*) forward repeats and between 16 (*D. genkwa)* and 25 (*D. odora*) palindrome repeats (Fig. S1). Only four reverse repeats were recorded, all in the plastome of *D. genkwa*, and no complementary repeats were detected.

### 3.3 Relative synonymous codon usage and predicted RNA editing sites

**Fig. 2:**
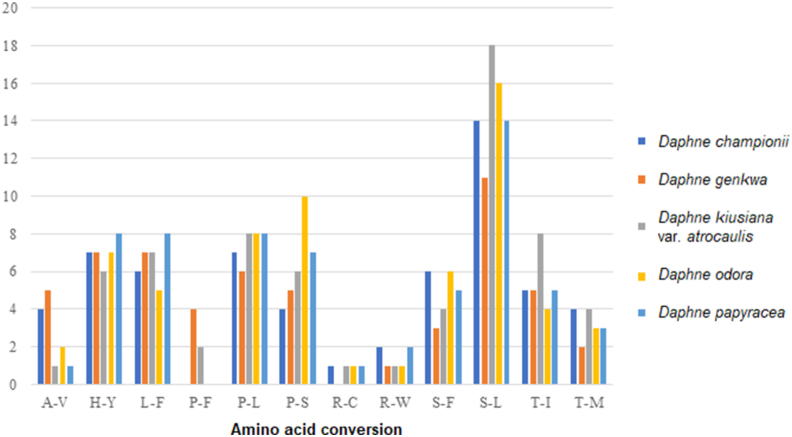
Amino acid conversions in potential RNA editing sites of the plastid genomes of five *Daphne* taxa, including alanine-to-valine (A-V), histidine-to-tyrosine (H-Y), leucine-to-phenylalanine (L-F), proline-to-phenylalanine (P-F), proline-to-leucine (P-L), proline-to-serine (P-S), arginine-to-cysteine (R-C), arginine-to-tryptophan (R-W), serine-to-phenylalanine (S-F), serine-to-leucine (S-L), threonine-to-isoleucine (T-I), and threonine-to-methionine (T-M).

The protein-coding sequences of *Daphne* plastomes had between 24,654 (*D. genkwa*) and 34,003 (*D. championii*) codons (Table S1). Among the encoded amino acids, leucine was most frequent in the plastomes of *D. genkwa*, *D. kiusiana* var. *atrocaulis*, and *D. odora*, and serine was most frequent in plastomes of *D. championii* and *D. papyracea*. While methionine was least frequent in the plastomes of *D. championii*, *D. odora*, and *D. papyracea*, tryptophan was the least frequent in the plastomes of *D. genkwa*, and *D. kiusiana* var. *atrocaulis*. Between 56 (*D. genkwa*) and 66 (*D. kiusiana*) potential RNA editing sites were predicted from the protein-coding genes of *Daphne* plastomes (Fig. 2). The most frequent amino acid conversion in all five *Daphne* species was serine-to-leucine (S-L). The least frequent amino acid conversions (i.e., occurred only once) differed among *Daphne* species. For instance, the least frequent amino acid conversion in *D. championii* was arginine-to-cysteine (R-C); in *D. genkwa* it was arginine-to-tryptophan (R-W). *D. kiusiana* var. *atrocaulis* had three one-time amino acid conversions (alanine-to-valine (A-V), R-C, and R-W), whereas *D. odora* (R-C and R-W) and *D. papyracea* (A-V and R-C) each had two.

### 3.4 Plastome variations

**Fig. 3:**
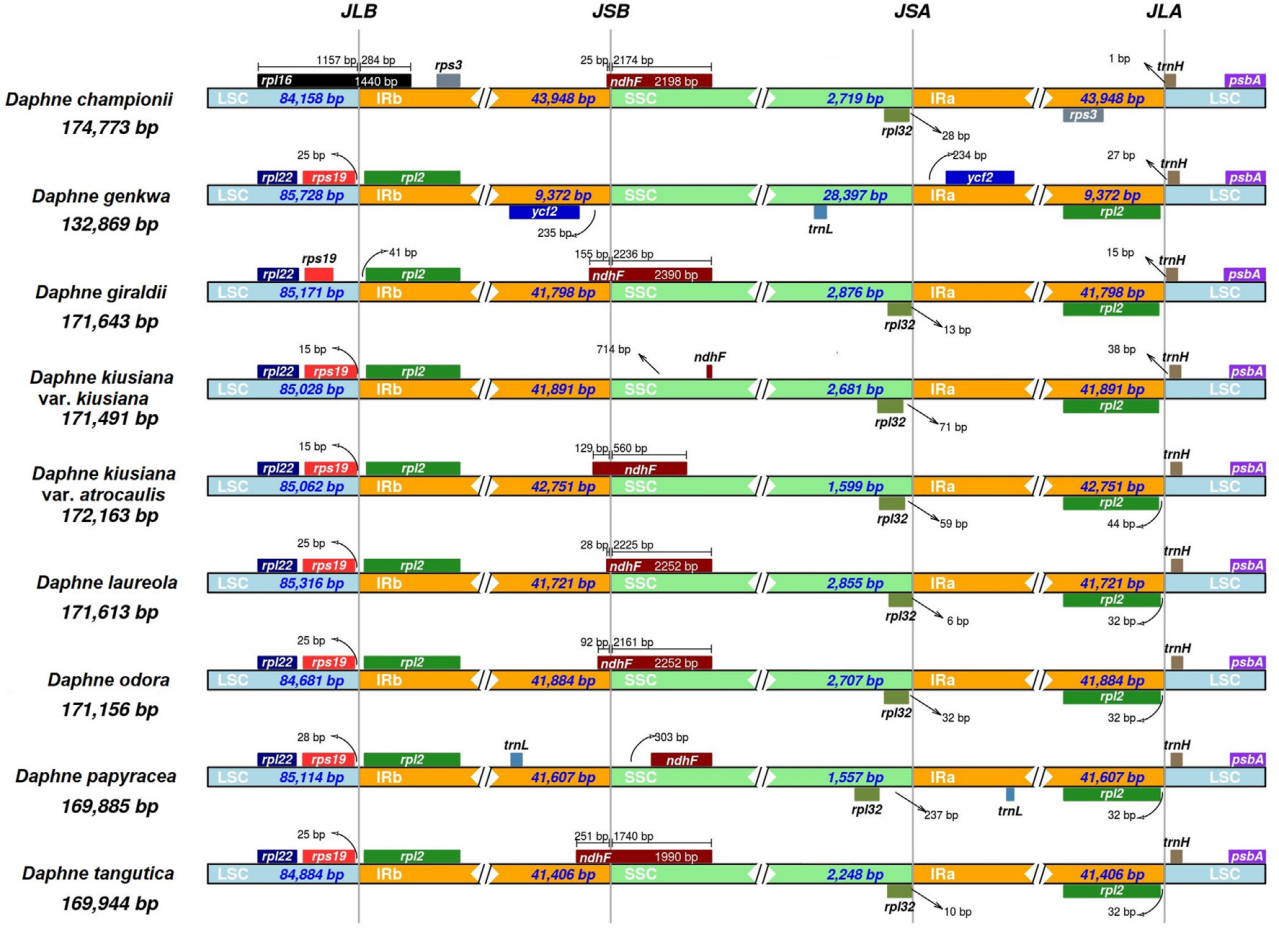
Inverted repeat (IR) border analysis based on the complete plastid genomes of nine *Daphne* taxa, including *D. championii*, *D. genkwa*, *D. giraldii*, *D. kiusiana* var. *kiusiana*, *D. kiusiana* var. *atrocaulis*, *D. laureola*, *D. odora*, *D. papyracea*, and *D. tangutica*.

Genetic pairwise distance based on plastome sequences of nine *Daphne* taxa was highest between *D. championii* and *D. genkwa* (0.0338), whereas the genetic distance was lowest between *D. kiusiana* var. *kiusiana* and *D. kiusiana* var. *atrocaulis* (0.0003) (Table 2). IR border analysis indicated that in all *Daphne* species except *D. championii*, *rps*19 and *rpl*2 genes were adjacent to the LSC/IRb junction (JLB); in *D. championii rpl*16 is located at the JLB (Fig. 3). *Ndh*F is located at the SSC/IRb junction (JSB) in all *Daphne* species except in three. In *D. kiusiana* var. *atrocaulis* and *D. papyracea*, the *ndh*F gene is located adjacent to the JSB; in *D. genkwa* the gene adjacent to the JSB is *ycf*2, which is located in the IRb region. In the other eight *Daphne* species the *ycf*2 gene is adjacent to the SSC/IRa junction (JSA) in the IRa region, whereas the *rpl*32 gene is adjacent to the JSA within the SSC. In all *Daphne* taxa, the *trn*H gene is located in the LSC region adjacent to the LSC/IRa junction (JLA). In *D. championii* the *rpl*2 gene located in the IRa region adjacent to the JLA is not present; instead the *rps*3 gene is located adjacent to the JLA.

**Table 2:** Interspecific pairwise distances of complete plastid genome sequences between nine *Daphne* taxa used in this study.

	DC	DGi	DKk	DKa	DP	DO	DT	DL
*Daphne championii* (DC)	–	–	–	–	–	–	–	–
*Daphne giraldii* (DGi)	0.0295	–	–	–	–	–	–	–
*Daphne kiusiana* var. *kiusiana* (DKk)	0.0288	0.0051	–	–	–	–	–	–
*Daphne kiusiana* var. *atrocaulis* (DKa)	0.0289	0.0052	0.0003	–	–	–	–	–
*Daphne papyracea* (DP)	0.0288	0.0051	0.0017	0.0018	–	–	–	–
*Daphne odora* (DO)	0.0286	0.0049	0.0016	0.0017	0.0016	–	–	–
*Daphne tangutica* (DT)	0.0285	0.0049	0.0015	0.0016	0.0014	0.0012	–	–
*Daphne laureola* (DL)	0.0286	0.0075	0.0069	0.0070	0.0068	0.0067	0.0066	–
*Daphne genkwa* (DGe)	0.0338	0.0229	0.0226	0.0226	0.0226	0.0224	0.0222	0.0220

**Fig. 4:**
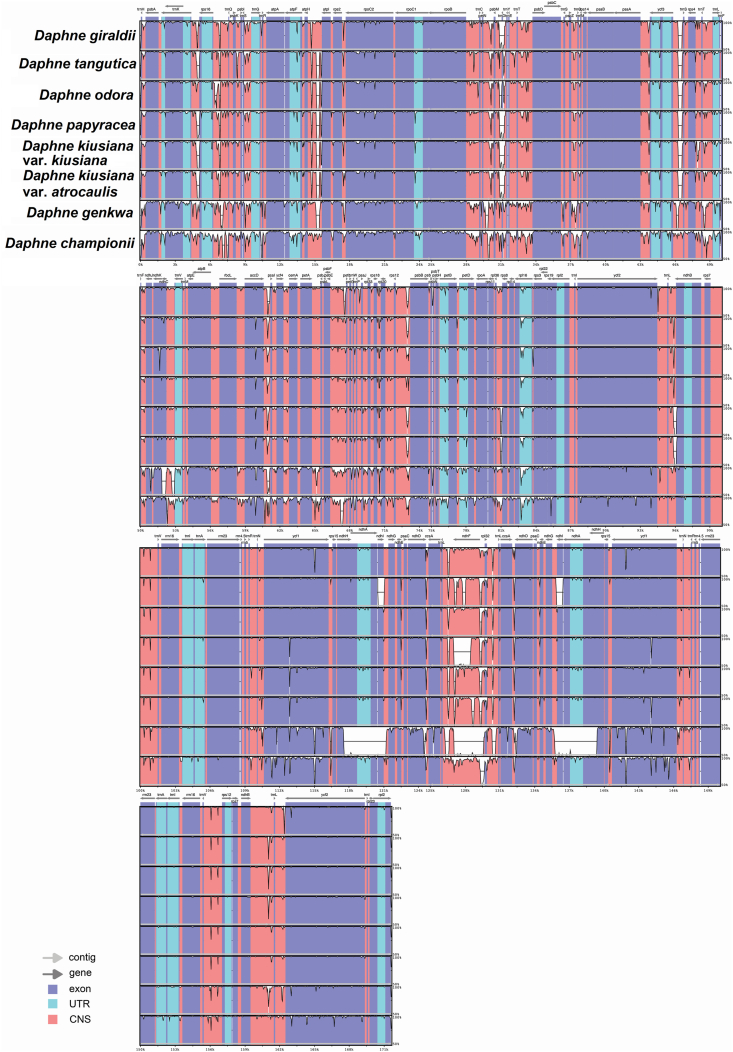
Comparative plastid genome analysis of nine *Daphne* taxa using mVISTA under Shuffle-LAGAN mode. Figure legend describes the direction and types of gene regions using color codes. Probability threshold was set at 50% and the plastid genome of *Daphne laureola* (MN201546) was selected as the reference genome.

The plastome alignment of nine *Daphne* taxa, with the plastome sequence of *D. laureola* as the reference genome, revealed high sequence conservatism across the plastomes of six *Daphne* taxa, including *D. giraldii*, *D. kiusiana* var. *atrocaulis*, *D. kiusiana* var. *kiusiana*, *D. odora*, *D. papyracea*, and *D. tangutica* (Fig. 4). Hyper-variable regions in the form of continuous distinct small gaps were detected in the LSC region of *D. championii* and *D. genkwa*, while three large gaps were detected in the SSC region of *D. genkwa*, when compared to *D. laureola*.

### 3.5 Phylogenetic inferences

**Fig. 5:**
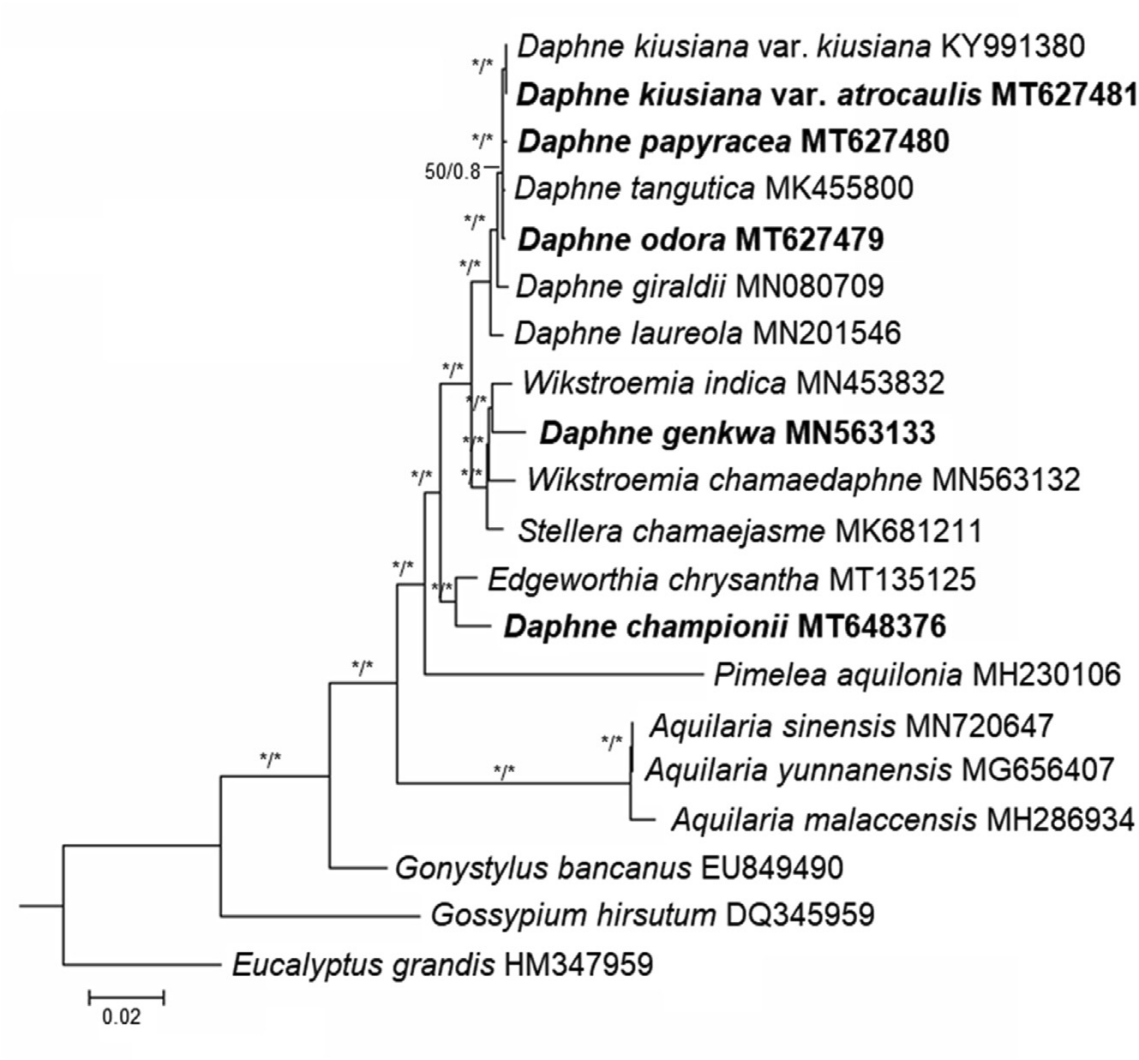
Phylogenetic relationships of *Daphne* and its allied genera in family Thymelaeaceae based on the plastid genome sequences of 18 taxa representing seven genera of the family Thymelaeaceae. The phylogenetic tree was constructed using both maximum likelihood (ML) and Bayesian inference (BI). All branch nodes were calculated with 1000 bootstrap replicates and reliable bootstrap supports (ML: BS ≥ 95%; BI: PP ≥ 90%) are indicated with an asterisk (∗). Sequences obtained through this study are indicated in bold and two species, *Eucalyptus grandis* (HM347959) and *Gossypium hirsutum* (DQ345959), were included as outgroups.

**Fig. 6:**
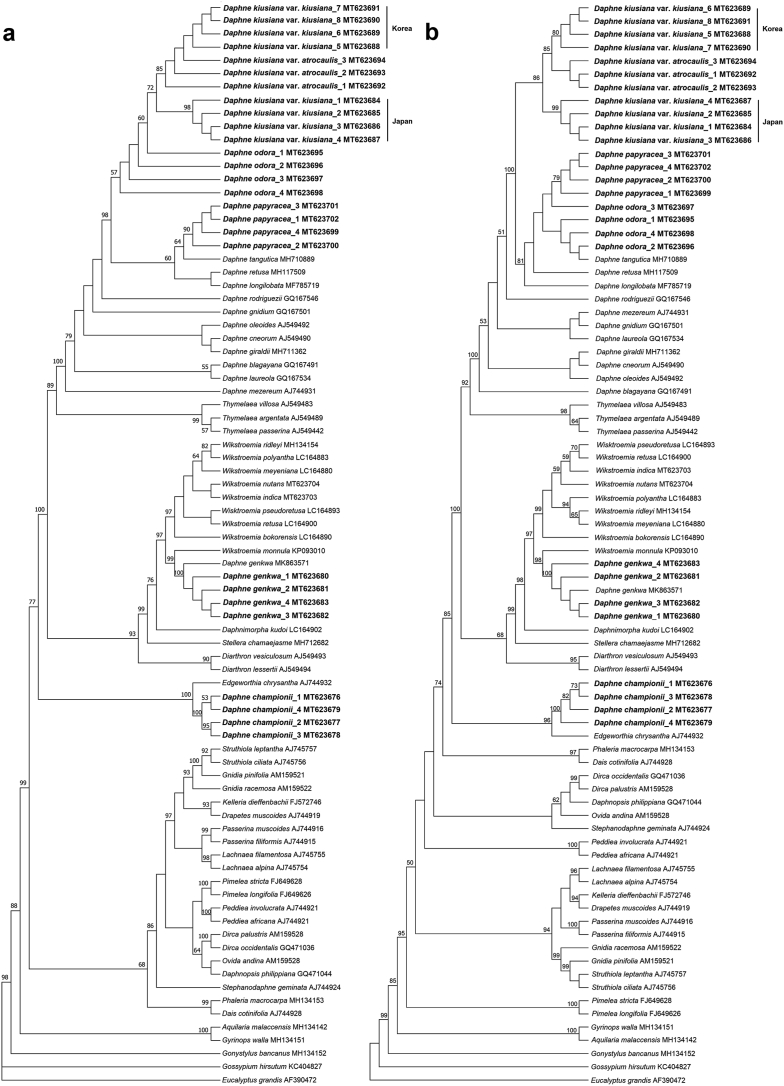
Phylogenetic analyses of family Thymelaeaceae based on the nuclear ribosomal internal transcribed spacer (nrITS) gene sequences of 58 taxa representing 24 genera from the family Thymelaeaceae. (a) maximum likelihood (ML), and (b) maximum parsimony (MP) tree analyses were conducted in 1000 bootstrap replicates. Trees are presented in consensus mode and sequences obtained through this study are indicated in bold. Only bootstrap support values that exceeded 50% are shown in both trees. Two species, *Eucalyptus grandis* (AF390472) and *Gossypium hirsutum* (KC404827), were included as outgroups.

ML and BI analyses based on the complete plastome sequences of nine *Daphne* taxa strongly suggested a paraphyletic relationship within *Daphne*, with three well-supported clades (BS ≥ 95%; PP ≥ 0.90) (Fig. 5). *Daphne championii* formed an independent clade and was sister to a species of another genus, *Edgeworthia chrysantha* Lindl. *D. genkwa* clustered with the *Wikstroemia* clade, sister to *Wikstroemia indica* (L.) C.A. Mey. *D. kiusiana* var. *atrocaulis*, *D. odora*, and *D. papyracea*, along with the four other *Daphne* species, formed a monophyletic group. ML and MP analyses based on nrITS sequences also revealed a paraphyletic relationship in *Daphne* (Fig. 6). The ML tree had a reliable backbone that was well-supported (BS ≥ 75%) for its major clades (Fig. 6a), but the major clades on the backbone of the MP tree was only partially supported (BS ≥ 75%) (Fig. 6b). Both ML and MP trees revealed similar clustering patterns for the five *Daphne* species. *D. championii* was sister to *E. chrysantha*; *D. genkwa* was nested within the *Wikstroemia* clade and was sister to *W. monnula* Hance; and *D. kiusiana* var. *atrocaulis*, *D. odora*, and *D. papyracea* formed a monophyletic clade with the other 12 *Daphne* species.

### 3.6 Divergence hotspots

**Fig. S2:**
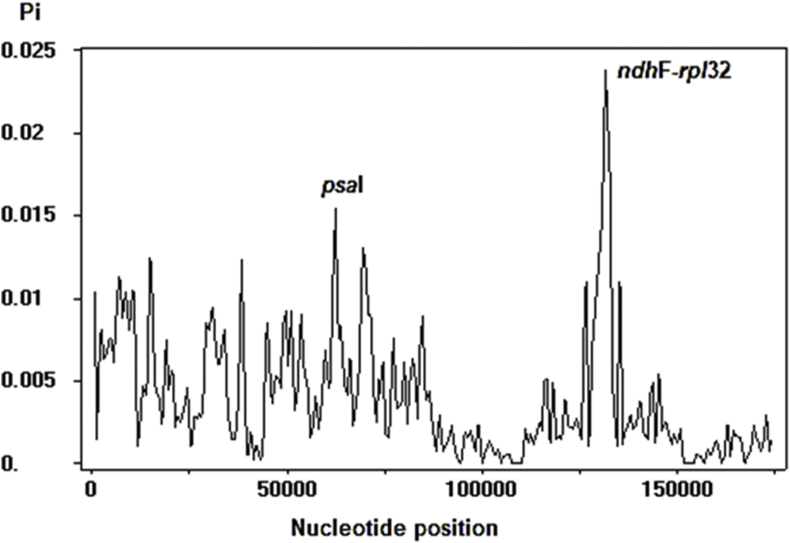
Nucleotide diversity (Pi) analysis based on the plastid genome sequences among seven selected *Daphne* taxa, excluding *D. championii* and *D. genkwa.*

Because our phylogenetic analysis placed *Daphne championii* and *D. genkwa* in unexpected positions, we excluded these species from our efforts to identify divergence hotspots. Genome alignment of seven species of *Daphne* indicated that Pi-values ranged from 0 to 0.02376 and had an average Pi-value of 0.00379. Two gene regions recorded Pi-values greater than our cut-off point (>0.015; Fig. S2). The two highly divergent regions were located at the *psa*I gene region (61,755 to 62,934 bp) of the LSC and the *ndh*F-*rpl*32 gene region (130,710 to 133,470 bp) of the SSC.

## 4 Discussion

### 4.1 Gene variations due to IR contraction and expansion

This study is the first to describe plastome sequences of *Daphne championii*, *D. genkwa*, *D. kiusiana* var. *atrocaulis*, *D. odora*, and *D. papyracea.* The total number of genes present in the plastomes ranges between 135 and 141 except for in *D. genkwa*, which has only 106. In addition, *D. genkwa* has an IR 4.5 times shorter than that of the average IR length in *Daphne* species. Furthermore, *D. genkwa* has an SSC region approximately 12 times longer than that of the average SSC of other *Daphne* taxa. Contraction and expansion at the IR borders are common during evolution and may cause variations in the size of each region or the plastome as a whole (Knox and Palmer, 1999). Analysis of gene order at the IR border allowed us to categorize the plastomes of the nine *Daphne* taxa examined into three types (Fig. 3) that we have named Type I, II, and III. Type I is the most common type and is found in the plastomes of *D. giraldii*, *D. kiusiana* var. *kiusiana*, *D. kiusiana* var. *atrocaulis*, *D. laureola*, *D. odora*, *D. papyracea*, and *D. tangutica*. Type II is exclusive to *D. genkwa*, whereas Type III is exclusive to *D. championii*. Type I and Type III plastomes have only two genes in the SSC region. In contrast, Type II plastomes have 20 genes in the SSC region. The plastomes of other members of Thymelaeaceae (e.g., *Aquilaria malaccensis* Lam., *E. chrysantha*, *Stellera chamaejasme* L., and *Wikstroemia chamaedaphne* (Bunge) Meisn.) exhibit gene content near the IR boundaries similar to that of Type I (Lee et al., 2018, Lee et al., 2020; Yun et al., 2019; Qian et al., 2020). However, due to the scarceness of Thymelaeaceae plastomes available in GenBank, we cannot conclude that Type I is the dominant gene order pattern at the IR borders.

### *4.2* Inter- and intraspecific plastome diversity of *Daphne kiusiana* complex

The taxonomic status of the *Daphne kiusiana* complex (*D. kiusiana* var. *kiusiana* and *D. kiusiana* var. *atrocaulis*) has been a long-standing debate. The *D. kiusiana* complex was initially treated as a variety of *D. odora* (Keissler, 1898; Rehder, 1916). However, it has since been proposed that *D. kiusiana* var. *atrocaulis* is closer to *D. papyracea* and *D. bholua* Buch.-Ham. ex D. Don than to *D. odora* and *D. kiusiana* var. *kiusiana* (Mathew, 1989). Taxonomic revisions of the *D. kiusiana* complex based on extensive morphological analysis have indicated that *D. kiusiana* var. *kiusiana* and *D. kiusiana* var. *atrocaulis* are distinct taxa (Wang et al., 2007a). We found that the inter- and intraspecific pairwise distance between the plastome sequences of *D. odora* and *D. kiusiana* complex were 0.0016 and 0.0017, respectively (Table 2). However, intraspecific pairwise analysis indicated that the genetic distance between *D. kiusiana* var. *kiusiana* and *D. kiusiana* var. *atrocaulis* was much smaller (0.0003). Although 64 singleton variable sites were detected between *D. kiusiana* var. *kiusiana* and *D. kiusiana* var. *atrocaulis* plastome sequences, the genetic distance between the two *D. kiusiana* varieties was much lower than the genetic distance between other species, e.g., *Cycas debaoensis* Y.C. Zhong & C.J. Chen (Cycadaceae; 0.0056) (Jiang et al., 2016). Moreover, the singletons found across two accessions of the same species were higher than those reported in the plastome sequences of *Camellia japonica* L. (Theaceae; 25 singletons) and *Dysphania pumilio* (R.Br.) Mosyakin & Clemants (Amranthaceae; 25 singletons) (Park and Kim, 2019; Park et al., 2019). This low nucleotide variation and small genetic distance are consistent with our observation that *D. kiusiana* var. *atrocaulis* is homogeneous with its original.

NrITS sequence analysis established that the genetic distance between *Daphne odora* and *D. kiusiana* var. *kiusiana* from Japan, *D. kiusiana* var. *kiusiana* from Korea, and *D. kiusiana* var. *atrocaulis* were 0.0040, 0.0013, and 0.0013 respectively. The genetic distance between *D. kiusiana* var. *atrocaulis* and each the Japanese *D. kiusiana* var. *kiusiana* and the Korean *D. kiusiana* var. *kiusiana* was 0.0026 for both accessions; the genetic distance between the Korean *D. kiusiana* var. *kiusiana* and *D. kiusiana* var. *atrocaulis* was zero. Five singleton variable sites were detected in the plastome alignment of three *D. kiusiana* accessions. All these sites were detected in sequences of two *D. kiusiana* accessions and the number of sites detected in the sequences for both the Japanese and Korean *D. kiusiana* var. *kiusiana* against *D. kiusiana* var. *atrocaulis* were four and one, respectively.

Phylogenetic analysis based on plastome sequences indicated that *Daphne kiusiana* var. *atrocaulis* is a well-supported sister to *D. kiusiana* var. *kiusiana* (Fig. 5); however, phylogenetic trees based on nrITS sequence data indicated that the intraspecific relationships are not well-defined. Our analysis did not consistently recognize *Daphne kiusiana* var. *kiusiana* as a monophyletic group, although samples from Korea and Japan were unequivocally established as two distinct monophyletic clades. Furthermore, MP and ML analyses did not establish *D. kiusiana* var. *atrocaulis* as monophyletic; also, the relationship between the *D. kiusiana* complex and *D. odora* was not uniform in either tree (Fig. 6). Discrepancies between the nrITS trees are likely because the computational approaches used for each model are influenced by evolutionary factors, including reversals, convergence, and homoplasy (Downie and Katz-Downie, 1996). The taxonomic status of the *D. kiusiana* complex derived from nuclear sequence data requires more extensive sampling and additional nuclear genes. Our phylogenetic analyses based on plastome sequences support morphological evidence that separates *D. kiusiana* from *D. odora*, although this finding is only partly supported by nrITS sequence data.

In our study, complete plastomes showed higher resolution in resolving species relationships than nrITS sequences (Fig. 5, Fig. 6). This implies that super-barcoding (or ultra-barcoding) of *Daphne* species using complete plastome sequences is reliable and effective (Kane et al., 2012; Li et al., 2015). However, although the cost of next-generation sequencing has become affordable in many countries, performing high-throughput DNA sequencing for diverse genera, such as *Daphne*, may be cost-prohibitive. Therefore, a cost-effective alternative such as using selected highly polymorphic and relatively shorter plastid barcodes is advisable. Previous studies suggested that identifying barcodes would require aligning eight to ten closely related plastome sequences from different species (Li et al., 2015), although five to seven plastomes have proven sufficient in many cases (Mwanzia et al., 2020; Liu et al., 2021). Accordingly, the *Daphne* DNA barcodes we identified, which are based on seven plastome sequences, should be reliable. Thus, we propose two potential DNA barcoding regions for *Daphne* species: the *psa*I gene and *ndh*F-*rpl*32 intergenic spacer region. DNA barcodes and gene markers derived from divergence hotspots in the plastome have been reported to be effective in identifying interrelated species within the plant kingdom (Li et al., 2015). To confirm the efficacy of these potential barcoding regions in delimitation and identification of *Daphne* species, their discrimination rate should be determined.

### 4.3 Current circumscription of the genus *Daphne* is polyphyletic

Molecular evidence based on both plastome and nrITS sequences clustered *Daphne genkwa* within the *Wikstroemia* clade and removed *D. championii* from the *Daphne* clade, placing it into an independent clade that has an affinity with *Edgeworthia chrysantha*. The phylogenetic positions of *D. championii* and *D. genkwa* have been a topic of debate for quite some time (Dute et al., 1996). Despite being under the same genus, the interspecific pairwise genetic distances between *D. championii* and *D. genkwa* were greater than those of other *Daphne* species (Table 2). The two species have long been considered morphologically, closely related and were grouped within the section *Genkwa* (Bentham and Hooker, 1880), a grouping that is still generally accepted. However, taxonomists have highlighted several morphological features in *D. championii* and *D. genkwa* that raised doubts about this treatment. For instance, the presence of long styles and filaments as well as short and upright calyx teeth suggest that *D. championii* should be grouped in the genus *Eriosolena* (Domke, 1934). *Daphne genkwa* exhibit the opposite leaf arrangement, which rarely occurs among *Daphne* species, and have disks at the base of the floral tube that divide into individual scales or threads, suggesting that the members of this genus should be reassigned to *Wikstroemia* (Domke, 1934).

The taxonomic delimitation of *Daphne* and *Wikstroemia* is challenging, with many *Wikstroemia* species previously known as members of *Daphne* and vice versa (Wang and Gilbert, 2007a; Wang et al., 2007a). Although the major morphological features that delimit *Daphne* from *Wikstroemia* are the shape of its disk and type of fruit, these features are not consistent across a number of species in either genus (Zhang et al., 2016). Furthermore, anatomical features, such as the presence of tori in the intervascular pit membrane (Dute et al., 1996) and leaf epidermal microfeatures (Zhang et al., 2015), are also unsuitable to delimit the two genera because tori are found in both genera and there is no significant variation in leaf epidermal microfeatures between members of *Daphne* and *Wikstroemia*. The *Daphne* section *Eriosolena*, which was initially proposed to belong to the genus *Daphne* (Bentham and Hooker, 1880), is now considered a distinct genus *Eriosolena* in the family Thymelaeaceae (Wang and Gilber, 2007b). Molecular evidence from our study showed that the strong sister relationship between *D. championii* and *E. chrysantha* has raised doubts about whether *D. championii* should be a member of *Eriosolena*. Researchers have proposed that *Daphne* can be delimited from the genera *Eriosolena* and *Edgeworthia* by the absence of bicollateral vascular bundles in their midribs (Domke, 1934). The close relationship between *Eriosolena* and *Edgeworthia* is supported by the absence of tori in *Eriosolena wallichii* Meisn., which was once considered a synonym of *Eriosolena involucrata* (Wall.) Tiegh (synonym *Eriosolena composita* (L.f.). Merr.) and is reported to have identical wood features as *Daphne pendula* Sm. (synonym *E. composita*) (The Plant List, 2010, The Plant List, 2013) and *Edgeworthia papyrifera* Siebold & Zucc. (synonym *E. chrysantha*) (Dute et al., 1996). Unfortunately, studies on the presence of tori in *D. championii* are not available and molecular information of the monotypic genus *Eriosolena* is scarce. Therefore, work to verify the presence of tori in *D. championii* and DNA sequencing of *E. composita* would be useful in providing insights to the taxonomic status of *D. championii*.

Taxonomic classification is primarily based on morphological characteristics (Greenman, 1940). However, our molecular data for *Daphne championii* and *D. genkwa* contradict morphology-based classifications. Specifically, we recommend that *D. championii* should be removed from *Daphne* and, after further study, *D. genkwa* should be reinstated to *Wikstroemia*.

### 4.4 *Daphne* and *Wikstroemia* are independent genera

The merging of *Wikstroemia* into *Daphne* was previously proposed based on traditional distinguishing characteristics (Halda, 2001); however, this treatment has not been universally accepted due to a lack of both morphological and molecular evidence, as well as the vast alteration on taxonomic nomenclature (Wang et al., 2007b; Zhang et al., 2007). Although our sample size was small, our results clearly demonstrate that *Daphne* and *Wikstroemia* are each monophyletic. Furthermore, molecular evidence in this study combined with previously identified morphological features (Domke, 1934; Dute et al., 1996) support the separation of *D. championii* and *D. genkwa* from *Daphne*. Taken together, these findings imply that the taxonomic confusion in *Daphne*, *Wikstroemia* and allied genera may have been caused by long-term misplacement of certain problematic taxa. If *Daphne* and *Wikstroemia* are not sister groups, as shown by both plastome and nrITS trees, the combination of both genera is not suitable and should be excluded. On the contrary, the independent taxonomic status of *Daphne* and *Wikstroemia* should be retained. Reasonable transfer of *Daphne* species to *Wikstroemia*, and vice versa, based on the morphological characteristics and molecular evidence will be appropriate under current genera delimitation.

Finally, we would like to iterate that evolutionary assessments based on limited sampling sizes, either molecular or morphological, can be elucidated when the context of other biological attributes is duly considered. However, further research that includes more taxa from Thymelaeaceae is essential to validate these hypotheses.

## Author contributions

SYL, WL and YZ conceived and designed this study. SYL, CH and JHL conducted the experiments. SYL and KX analyzed the data. SYL wrote the manuscript. KX, JHL, WL, YZ and QF edited the manuscript. All authors read and approved the manuscript.

## Declaration of competing interest

The authors declare that they have no conflict of interest.
