# Supplementary material for: Molecular phylogenetic analyses based on the complete plastid genomes and nuclear sequences reveal Daphne (Thymelaeaceae) to be non-monophyletic as current circumscription
Source: Plant Divers. 2021 Nov 11;44(3):279–89. doi: 10.1016/j.pld.2021.11.001 (PMC9209861; doi:10.1016/j.pld.2021.11.001)
Supplement: Multimedia component 1 [file mmc1.docx]

**Table S1** Relative synonymous codon usage of amino acids in *Daphne championii*, *D. genkwa*, *D. kiusiana* var. *atrocaulis*, *D. odora*, and *D. papyracea* plastid genomes.

| **Amino acid** | **Codon** | ***Daphne championii*** | | ***Daphne genkwa*** | | ***Daphne kiusiana var. atrocaulis*** | | ***Daphne odora*** | | ***Daphne papyracea*** | |
| --- | --- | --- | --- | --- | --- | --- | --- | --- | --- | --- | --- |
|  |  | **Quantity** | **RSCU** | **Quantity** | **RSCU** | **Quantity** | **RSCU** | **Quantity** | **RSCU** | **Quantity** | **RSCU** |
| Alanine (Ala) | GCG | 163 | 0.76 | 103 | 0.68 | 161 | 0.8 | 174 | 0.64 | 160 | 0.6 |
|  | GCA | 265 | 1.2 | 202 | 1.36 | 251 | 1.28 | 336 | 1.24 | 333 | 1.2 |
|  | GCT | 252 | 1.16 | 175 | 1.16 | 212 | 1.08 | 384 | 1.4 | 391 | 1.44 |
|  | GCC | 198 | 0.92 | 121 | 0.8 | 163 | 0.84 | 204 | 0.76 | 211 | 0.76 |
| Cystine (Cys) | TGT | 471 | 1.1 | 302 | 1.02 | 424 | 1.1 | 437 | 1.18 | 443 | 1.18 |
|  | TGC | 380 | 0.9 | 291 | 0.98 | 342 | 0.9 | 305 | 0.82 | 309 | 0.82 |
| Asparticacid (Asp) | GAT | 703 | 1.42 | 436 | 1.36 | 629 | 1.4 | 791 | 1.48 | 773 | 1.48 |
|  | GAC | 283 | 0.58 | 202 | 0.64 | 274 | 0.6 | 278 | 0.52 | 270 | 0.52 |
| Glutamicacid (Glu) | GAG | 430 | 0.66 | 346 | 0.72 | 442 | 0.72 | 400 | 0.58 | 374 | 0.56 |
|  | GAA | 857 | 1.34 | 611 | 1.28 | 786 | 1.28 | 997 | 1.42 | 965 | 1.44 |
| Phenylalanine (Phe) | TTT | 1524 | 1.28 | 952 | 1.14 | 1423 | 1.22 | 1588 | 1.32 | 1505 | 1.3 |
|  | TTC | 851 | 0.72 | 708 | 0.86 | 903 | 0.78 | 828 | 0.68 | 826 | 0.7 |
| Glycine (Gly) | GGG | 467 | 1.12 | 359 | 1.16 | 434 | 1.16 | 432 | 0.96 | 442 | 0.96 |
|  | GGA | 566 | 1.36 | 382 | 1.24 | 498 | 1.32 | 683 | 1.52 | 709 | 1.52 |
|  | GGT | 377 | 0.88 | 270 | 0.88 | 337 | 0.88 | 449 | 1 | 447 | 0.96 |
|  | GGC | 274 | 0.64 | 212 | 0.68 | 248 | 0.64 | 231 | 0.52 | 253 | 0.56 |
| Histidine (His) | CAT | 558 | 1.44 | 367 | 1.36 | 481 | 1.32 | 588 | 1.44 | 583 | 1.44 |
|  | CAC | 213 | 0.56 | 169 | 0.64 | 243 | 0.68 | 227 | 0.56 | 224 | 0.56 |
| Isoleucine (Ile) | ATA | 710 | 0.81 | 628 | 0.93 | 751 | 0.87 | 740 | 0.87 | 717 | 0.84 |
|  | ATT | 1215 | 1.38 | 834 | 1.23 | 1065 | 1.23 | 1200 | 1.41 | 1198 | 1.41 |
|  | ATC | 738 | 0.84 | 568 | 0.84 | 761 | 0.9 | 633 | 0.75 | 610 | 0.72 |
| Lysine (Lys) | AAG | 702 | 0.66 | 602 | 0.74 | 791 | 0.72 | 617 | 0.6 | 587 | 0.58 |
|  | AAA | 1407 | 1.34 | 1011 | 1.26 | 1434 | 1.28 | 1470 | 1.4 | 1416 | 1.42 |
| Leucine (Leu) | TTG | 733 | 1.38 | 624 | 1.44 | 803 | 1.44 | 712 | 1.32 | 654 | 1.32 |
|  | TTA | 745 | 1.44 | 524 | 1.2 | 678 | 1.2 | 812 | 1.56 | 767 | 1.56 |
|  | CTG | 265 | 0.48 | 265 | 0.6 | 326 | 0.6 | 251 | 0.48 | 212 | 0.42 |
|  | CTA | 443 | 0.84 | 423 | 0.96 | 551 | 0.96 | 465 | 0.9 | 407 | 0.84 |
|  | CTT | 635 | 1.2 | 501 | 1.14 | 659 | 1.14 | 620 | 1.2 | 605 | 1.26 |
|  | CTC | 320 | 0.6 | 256 | 0.6 | 377 | 0.66 | 305 | 0.6 | 275 | 0.54 |
| Methoinine (Met) | ATG | 615 | 1 | 533 | 1 | 679 | 1 | 575 | 1 | 537 | 1 |
| Asparagine (Asn) | AAT | 1268 | 1.4 | 832 | 1.36 | 1130 | 1.34 | 1115 | 1.38 | 1124 | 1.38 |
|  | AAC | 550 | 0.6 | 396 | 0.64 | 565 | 0.66 | 500 | 0.62 | 513 | 0.62 |
| Proline (Pro) | CCG | 219 | 0.8 | 186 | 0.84 | 259 | 0.96 | 214 | 0.72 | 188 | 0.68 |
|  | CCA | 321 | 1.16 | 294 | 1.32 | 339 | 1.24 | 326 | 1.12 | 325 | 1.16 |
|  | CCT | 300 | 1.08 | 223 | 1 | 263 | 0.96 | 387 | 1.32 | 369 | 1.32 |
|  | CCC | 261 | 0.96 | 178 | 0.8 | 220 | 0.8 | 239 | 0.8 | 229 | 0.84 |
| Glutamine (Gln) | CAG | 311 | 0.64 | 263 | 0.7 | 323 | 0.68 | 281 | 0.52 | 261 | 0.52 |
|  | CAA | 671 | 1.36 | 490 | 1.3 | 633 | 1.32 | 787 | 1.48 | 760 | 1.48 |
| Arginine (Arg) | AGG | 515 | 1.26 | 381 | 1.32 | 476 | 1.26 | 408 | 1.08 | 406 | 1.08 |
|  | AGA | 896 | 2.22 | 552 | 1.92 | 803 | 2.16 | 858 | 2.22 | 862 | 2.22 |
|  | CGG | 276 | 0.66 | 251 | 0.9 | 296 | 0.78 | 242 | 0.6 | 220 | 0.6 |
|  | CGA | 422 | 1.02 | 261 | 0.9 | 347 | 0.96 | 411 | 1.08 | 416 | 1.08 |
|  | CGT | 193 | 0.48 | 144 | 0.48 | 151 | 0.42 | 258 | 0.66 | 267 | 0.72 |
|  | CGC | 146 | 0.36 | 127 | 0.42 | 141 | 0.36 | 138 | 0.36 | 142 | 0.36 |
| Serine (Ser) | AGT | 470 | 0.84 | 272 | 0.78 | 427 | 0.9 | 488 | 0.96 | 504 | 0.96 |
|  | AGC | 412 | 0.78 | 260 | 0.78 | 337 | 0.72 | 348 | 0.66 | 367 | 0.72 |
|  | TCG | 438 | 0.78 | 266 | 0.78 | 418 | 0.84 | 382 | 0.72 | 380 | 0.72 |
|  | TCA | 691 | 1.26 | 473 | 1.38 | 627 | 1.26 | 680 | 1.32 | 692 | 1.32 |
|  | TCT | 775 | 1.44 | 462 | 1.38 | 697 | 1.44 | 727 | 1.44 | 718 | 1.38 |
|  | TCC | 477 | 0.9 | 306 | 0.9 | 421 | 0.84 | 457 | 0.9 | 465 | 0.9 |
| Threonine (Thr) | ACG | 241 | 0.68 | 183 | 0.76 | 250 | 0.76 | 231 | 0.68 | 225 | 0.64 |
|  | ACA | 455 | 1.24 | 333 | 1.36 | 418 | 1.28 | 439 | 1.28 | 432 | 1.24 |
|  | ACT | 441 | 1.24 | 266 | 1.08 | 335 | 1.04 | 429 | 1.24 | 420 | 1.2 |
|  | ACC | 308 | 0.84 | 206 | 0.84 | 285 | 0.88 | 294 | 0.84 | 303 | 0.88 |
| Valine (Val) | GTG | 239 | 0.72 | 229 | 0.84 | 282 | 0.76 | 210 | 0.6 | 190 | 0.6 |
|  | GTA | 388 | 1.12 | 341 | 1.24 | 418 | 1.16 | 432 | 1.28 | 405 | 1.28 |
|  | GTT | 500 | 1.48 | 358 | 1.28 | 479 | 1.32 | 497 | 1.44 | 471 | 1.48 |
|  | GTC | 237 | 0.68 | 184 | 0.68 | 268 | 0.76 | 224 | 0.64 | 212 | 0.68 |
| Tryptophan (Trp) | TGG | 641 | 1 | 489 | 1 | 620 | 1 | 674 | 1 | 649 | 1 |
| Tyrosine (Tyr) | TAT | 1140 | 1.4 | 679 | 1.3 | 1040 | 1.38 | 1092 | 1.42 | 1059 | 1.4 |
|  | TAC | 486 | 0.6 | 360 | 0.7 | 475 | 0.62 | 448 | 0.58 | 446 | 0.6 |
| End | TGA | 762 | 1.17 | 460 | 0.96 | 723 | 1.05 | 653 | 1.11 | 677 | 1.2 |
|  | TAG | 510 | 0.78 | 460 | 0.96 | 585 | 0.87 | 466 | 0.78 | 416 | 0.72 |
|  | TAA | 683 | 1.05 | 512 | 1.08 | 731 | 1.08 | 650 | 1.11 | 617 | 1.08 |
